# Research on Calf Behavior Recognition Based on Improved Lightweight YOLOv8 in Farming Scenarios

**DOI:** 10.3390/ani15060898

**Published:** 2025-03-20

**Authors:** Ze Yuan, Shuai Wang, Chunguang Wang, Zheying Zong, Chunhui Zhang, Lide Su, Zeyu Ban

**Affiliations:** 1 College of Electromechanical Engineering, Inner Mongolia Agricultural University, Hohhot 010018, China; nmgnydxyz@emails.imau.edu.cn (Z.Y.); jdwangshuai@163.com (S.W.); jdwcg@imau.edu.cn (C.W.); zhangchunhui@imau.edu.cn (C.Z.); sld@imau.edu.cn (L.S.); banzeyu0206@163.com (Z.B.); 2 The Innovation Team of Higher Education Institutions in Inner Mongolia Autonomous Region, Hohhot 010018, China; 3 Inner Mongolia Engineering Research Center of Intelligent Equipment for the Entire Process of Forage and Feed Production, Hohhot 010018, China; 4 Full Mechanization Research Base of Dairy Farming Engineering and Equipment, Ministry of Agriculture and Rural Affairs of the People’s Republic of China, Hohhot 010018, China

**Keywords:** calf, behavior recognition, lightweighting, pruning, image detection

## Abstract

In order to achieve accurate and efficient recognition of calf behavior in complex scenes such as cow overlapping, occlusion, and different light and occlusion levels, this experiment adopts the method of improving the YOLO v8 model to recognize calf behavior. A calf daily behavior dataset containing 2918 images is selected as the test benchmark through video frame extraction; a P2 small-target detection layer is introduced to improve the resolution of the input scene, which significantly improves the model recognition accuracy, and reduces the computational complexity and storage requirements of the model through the Lamp pruning method. Comparisons are made with the SSD, YOLOv5n, YOLOv8n, YOLOv8-C2f-faster-EMA, YOLO v11n, YOLO v12n, and YOLO v8-P2 advanced models. The results show that the number of parameters, floating point operations (FLOPs), model size, and mean average precision (mAP) of the model after introducing the P2 small-target detection layer and pruning with the Lamp strategy are 0.949 M, 4.0 G, 2.3 Mb, and 90.9%, respectively. The significant improvement in each index effectively reduces the model size and improves the accuracy of the network. The detection results in complex environments with different light and shading levels show that the mAP in daytime (exposure) and nighttime environments is 85.1% and 84.8%, respectively, and the average mAP in the three kinds of shading cases (light, medium, and heavy) is 87.3%, representing a lightweight, high-precision, real-time, and robust model. The results of this study provide a reference for the real-time monitoring of calf behaviors all day long in complex environments.

## Simple Summary

To address the scarcity of specialized datasets for calf behavior analysis, this study constructed a comprehensive dataset comprising 2918 images capturing five key behaviors, namely walking, standing, lying, feeding, and drinking, under diverse environmental conditions, including varying light intensities, such as daytime, nighttime, and overexposure, and light, moderate, and heavy occlusion levels. Building upon YOLOv8, we introduced a lightweight architecture, YOLOv8-P2-Lamp, incorporating two critical enhancements: a P2 small-target detection layer to improve recognition of subtle features such as leg articulation, and the Lamp pruning strategy to eliminate redundant channels while preserving accuracy. The optimized model achieved a 90.9% mAP with only 0.949 M parameters, 4.0 G FLOPs, and a 2.3 MB model size. Finally, it was compared with several advanced models in complex environments with different light intensities and different occlusion situations to summarize and analyze it.

## 1. Introduction

In recent years, with the improvement in residents’ living standards, there has been a significant increase in dietary nutritional demands. Dairy products, due to their high nutritional value, have become increasingly important in daily life [1,2]. However, according to farming experience, more than half of cow mortality events occur during the calf period, especially during the newborn calf stage [3,4,5]. Additionally, diseases during the calf period have been shown to reduce average daily weight gain and increase the risk of illness [6,7,8]. Healthy calves are the cornerstone of the cattle production system. To reduce mortality, improve health, and enhance performance, it is crucial to observe abnormal behaviors in calves during illness [9,10,11].

Traditional monitoring and evaluation methods often require manual intervention, are time-consuming, and are prone to errors, making them unsuitable for meeting the high-efficiency and large-scale needs of the livestock industry [12,13,14]. As a result, researchers at home and abroad have focused on animal behavior recognition methods, mainly divided into contact-based sensor monitoring and non-contact sensor monitoring of animal behavior. Currently, sensor-based contact methods are used for beef cattle behavior recognition. This method requires sensors to be installed on different parts of cattle, and behavior is determined based on the collected data, such as activity level, temperature, and sound. However, this approach can cause stress responses in cattle, and the high probability of sensor damage may adversely affect the accuracy of behavior recognition [15,16,17]. Peng Y et al. [18] installed Inertial Measurement Unit (IMU) collars on dairy cows that were about to give birth, collecting and classifying data. The LSTM-RNN model was used for training to classify dairy cow behavior. The results demonstrated the potential of the model to automatically recognize behaviors before birth.

In the field of animal behavior recognition, commonly used non-contact sensors include thermal infrared sensors and 2D and 3D cameras. Ma et al. [19] implemented a Rexnet 3D network pair to achieve non-contact automatic recognition of basic locomotor behaviors in dairy cows. The results showed that the accuracy of behavior recognition with this method was 95.00% and 91.02% when the unedited video was tested with a sampling interval of five frames, which proved that this network can provide a reference for accurate breeding and health welfare of dairy cows. Yin et al. [20] proposed a dairy cow behavior recognition technique based on EficientNet-LSTM by introducing a bidirectional feature pyramid network (BiFPN) to optimize EficientNet to achieve feature aggregation, to optimize the bidirectional long-term and short-term memory network with the help of an attention mechanism, and to finally achieve the recognition of five behaviors of a single cow, namely walking, standing, lying, lactating, and drinking, with a recognition accuracy of 95.2%. Wang Fei et al. [21] proposed a fast automatic detection method based on a real-time multi-objective attention model for multiple parts of cows, which can eliminate the influence of light, overlap, multiple types, etc., on the image detection results in the existing image detection of cows, and then realize the accurate classification and recognition of multi-scale and multi-parts of the state information of cows. The improved model SNSS-YOLO v7 proposed by Qingling Duan et al. [22] is able to identify seven behaviors of cattle herds of different densities with an average accuracy of 95.2%, and the memory footprint of the improved model is only 39 MB. The advantage of these sensors is that they are flexible to install and use, and they do not damage or interfere with the cattle, which reduces the stress reaction and protects animal welfare.

Improving deep learning models by replacing and adding network modules alone makes it difficult to achieve a balance between significantly reducing the model size and ensuring the model accuracy, which is the main reason why the non-contact monitoring technology is limited to laboratory use and difficult to deploy in real-world scenarios. Therefore, this paper proposes a lightweight calf behavior recognition algorithm based on the improved YOLO v8-P2 model. The main contributions of this paper are shown in Table 1.

This paper is structured as follows: Section 1 introduces the data acquisition and preprocessing methods; Section 2 presents the improved YOLOv8-P2-Lamp model; Section 3 compares and analyzes the experimental results; Section 4 discusses the model limitations; and Section 5 summarizes the full paper.

## 2. Materials and Methods

### 2.1. Data Sources

**Figure 1:**
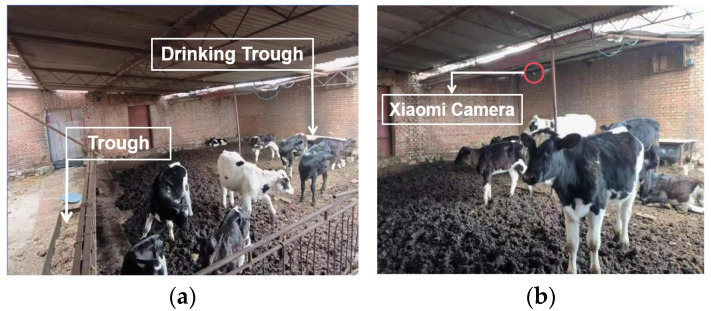
Calf test site. (**a**) Example of barn environment; (**b**) camera installation location.

The experimental data for this study were collected from September 2023 to September 2024. The site is a small-scale farm located in a cattle barn in Tumote Right Banner, Inner Mongolia Autonomous Region. The calf activity area is a fixed range in the pen, 10 m long and 7 m wide, with cameras mounted in the corners of the area to cover the entire activity space. A Xiaomi 2K version PTZ camera (Xiaomi Technology, Shanghai, China) was installed in the corner of the shelter in the activity area, at a height of about 3 m, with a downward viewing angle of approximately 30°. The barn environment is shown in Figure 1, where Figure 1a represents the barn environment, and Figure 1b shows the camera installation location.

### 2.2. Dataset Production

This study focused on 14 Holstein calves, with a total of 988 video segments containing calf behavior data. The dataset includes 950 short videos of approximately 5 min each and 38 long videos ranging from 0.5 to 3 h. The video resolution is 1920 × 1080 pixels, and the frame rate is 25 fps. The behavior data include multiple scenes, angles, and lighting conditions, reflecting the characteristics of a confined farming environment.

To reduce redundant frames in the dairy cow behavior detection dataset, this study used unsupervised knowledge distillation (Distillation with No Labels, DINO), Equation (1), and the K-means clustering algorithm (K-means), Equation (2), to extract the feature vector for each frame and perform K-means clustering on the feature matrix of the images. Finally, a representative image was selected from each cluster to complete the deduplication process using Equation (3).

(1) $$f_{i}=DINO\left(x_{i}\right)$$

(2) $$J=\sum_{k}^{K}\;\sum_{x_{i}\in C_{k}}\;∥f_{i}-\mu_{k}{∥}^{2}$$

(3) $$x_{k}^{*}=arg\underset{x_{i}\in C_{k}}{\min}d\left(f_{i},\mu_{k}\right)$$

**Figure 2:**
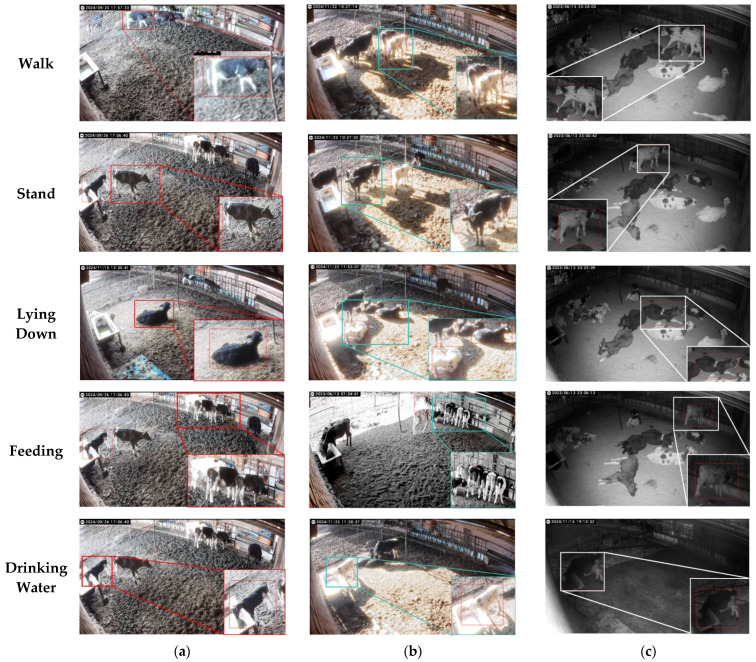
Examples of calf behavior shots. (**a**) Examples of calf behavior during the day; (**b**) examples of calf behavior during the day (against the light); (**c**) examples of calf behavior at night. The information inside the boxes provides close-up images of the calves under different lighting conditions.

In these equations, $f_{i}$ represents the high-dimensional feature vector of the image, K is the number of clusters, $C_{k}$ is the K-th cluster, $\mu_{k}$ is the cluster center, and $x_{k}^{*}$ is the representative image of the K-th cluster. After deduplicating the images, the calf behavior dataset was split into a training set and a test set in an 8:2 ratio, resulting in 2334 training images and 584 test images. A sample of the data is shown in Figure 2, which illustrates mixed behaviors such as walking and standing under different lighting intensities. The video collection period was from 10:00 a.m. to 1:00 a.m., with the period from 10:00 a.m. to 6:00 p.m. defined as daytime and that from 6:00 p.m. to 1:00 a.m. defined as nighttime.

In this study, five categories of behavioral traits were defined based on calf behavioral criteria in collaboration with livestock experts from Inner Mongolia Agricultural University—walking, standing, lying down, eating, and drinking—as the research subjects. The behavior determination criteria for dairy cows were set as shown in Table 2. Table 2 defines the five behaviors of calves and their corresponding data information. When walking, the calf alternates the bending of its limbs, keeping the torso horizontal and the head slightly raised, with the abdomen away from and parallel to the ground. In contrast, when standing, the limbs are erect and perpendicular to the ground, with no bending of the legs. When eating, the calf stands with its limbs straight and its head lowered and passing through the barriers, with its mouth near the feed. The calf uses its tongue to scoop up the food, accompanied by licking or chewing behaviors. When drinking, the calf approaches the water trough, with its head slightly raised above the trough, exhibiting intermittent head movements and looking around. When lying down, the calf’s body is close to the ground, with its abdomen in contact with the floor, its limbs bent, and its posture relaxed, which is distinctly different from the other behaviors.

**Figure 3:**
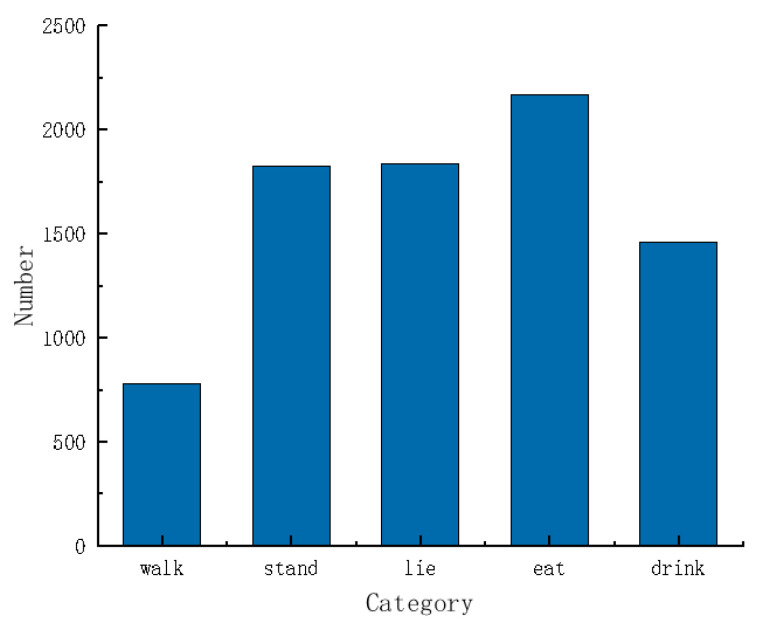
Behavioral labeling analysis of cows.

Based on the definitions in Table 1, a total of 8064 behavior labels were obtained, with 778 for walking, 1824 for standing, 1834 for lying, 2170 for eating, and 1458 for drinking. Figure 3 shows the comparative statistical analysis of the label quantities. Among these, the eating behavior had the highest number of occurrences, with standing and lying behaviors having similar quantities. Drinking behavior was less frequent, and walking behavior data made up the smallest proportion. These proportions align with the behavior distribution of cows in real farming environments, fulfilling the requirement for calf behavior distribution under different conditions.

### 2.3. Test Platform and Model Test Index

#### 2.3.1. Test Environment and Parameter Setting

In this study, the computational platform used was configured as follows: The processor was an Intel(R) Xeon(R) Gold 6248R CPU (Intel Corporation, Santa Clara, CA, USA) @ 3.00 GHz with a 2.99 GHz clock speed. The system was equipped with 64 GB of RAM, 8 TB of SSD storage, and an NVIDIA GeForce RTX A6000 GPU with 48 GB of video memory (NVIDIA Corporation, Santa Clara, CA, USA). The software environment included the Windows 11 operating system with CUDA 12.1 installed, as well as Python 3.8. The deep learning framework used for this experiment was Pytorch version 1.12.1. All comparative algorithms were executed in the same environment. In this study, the parameter configurations in the literature [23,24] were referenced, as shown in Table 3.

#### 2.3.2. Test Evaluation Indicators

To verify the performance of the improved model, this study evaluated the model using six metrics: precision (P), recall (R), average precision (AP), mean average precision (mAP), number of parameters (Params), and floating point operations (FLOPs). mAP50 refers to calculating the average precision (AP) for each class with a threshold of 0.5 and then averaging the AP across all classes. The calculations for P, R, and mAP are as shown in Equations (4)–(6) [25].

(4) $$P=\frac{TP}{\left(TP+FP\right)}\times 100\%$$

(5) $$R=\frac{TP}{\left(TP+FN\right)}\times 100\%$$

(6) $$mAP=\frac{\sum_{i=1}^{C}AP(C)}{C}\times 100\%$$

In the formula, TP represents the number of instances that are actually positive and classified as positive, FP represents the number of instances that are actually negative but classified as positive, FN represents the number of instances that are actually positive but classified as negative, and C represents the number of detection categories. In this study, C = 1.

### 2.4. YOLOv8n-P2-Lamp Lightweight Calf Behavior Recognition Model

**Figure 4:**
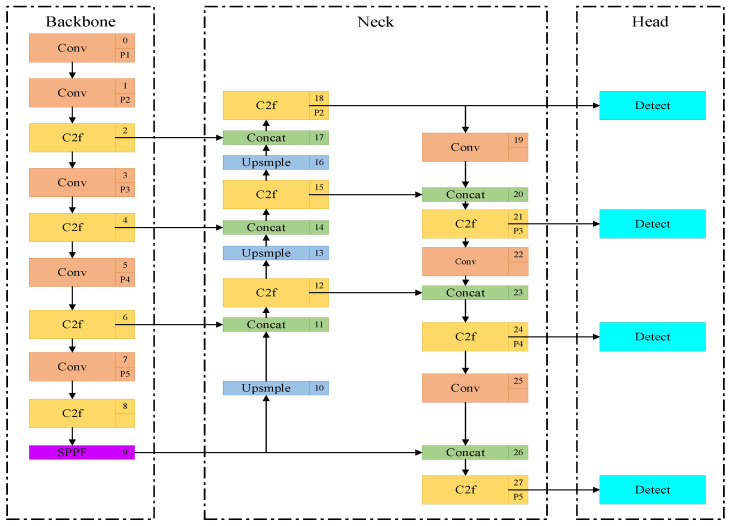
YOLO v8n-P2 network modeling.

In order to improve the differentiation between the standing and walking behaviors of calves, the existing YOLO v8n model was improved in this study. Through observation, in the daily activities of calves, due to the fact that the legs occupy fewer pixels in the image, the subtle difference in the degree of leg bending often leads to difficulty in distinguishing these two behaviors, which can be easily ignored or misjudged by the model. To address this problem, this paper introduces a P2 small-target detection layer into the Head layer of the original model. This improvement enables the YOLO v8 model to capture the leg features in more detail, especially the subtle differences in the degree of leg bending, and thus distinguish standing and walking behaviors more effectively. This optimization resulted in a significant improvement in the detection accuracy of the model, providing more reliable technical support for calf behavior analysis. The improved network structure is shown in Figure 4.

### 2.5. P2 Detection Layer

One of the reasons for the poor detection effect of YOLO v8 may be that the behavioral action amplitude is small, while the downsampling multiplier of yolov8 is relatively large, and it is difficult for the deeper feature maps to learn the feature information of the target; therefore, it is proposed to add a target detection layer to the detection of shallower feature maps spliced with the deeper feature maps, so that the network pays more attention to the detection of the fine action and improves the detection effect.

**Figure 5:**
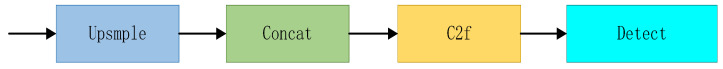
P2 detection layer.

In the target detection framework of YOLOv8, the feature pyramid network (FPN) [26] plays an important role. The P2 layer, as part of the FPN, plays an important role in the process of multi-scale feature fusion. When dealing with calf behavioral features, duplicated or unnecessary data may be encountered, for example, variable light, body contact, and cross-obscuration between calves. Calf behavioral postures are also varied, and each posture requires different features to describe it, resulting in an increase in the dimensionality of the data. All of these factors may increase the complexity of the model and the redundancy of the data features. In order to solve this problem and enhance the feature processing capability of the model, this study introduces a lightweight and high-precision P2 small-target detection layer and constructs a new feature extraction C2f-Faster module. The specific structure of the P2 small-target detection layer is shown in Figure 5, where there is an Upsmple layer for each Detect output layer, and then the Concat module is used to integrate the Backbone network. The two Cony layers together form the structure of the inverse residual block, and its middle layer has an extended number of channels, on which shortcut connections are added to reuse the input features. The C2f-Faster structure is based on the C2f structure, which divides the input data through the Conv layer into two branches for processing. One branch is passed directly to the feature connection module Concat, and the other branch is processed by the FasterNet Block. Finally, the results of the two parts are spliced in the channel dimension, and the output results are obtained through the Cony layer. Embedding the FasterNet Block into the C2f structure allows the C2f-Faster module to learn rich features while reducing the computational load and complexity of the model.

### 2.6. P2 Channel Pruning

Through network improvements, the accuracy of the model was enhanced. To further improve the model’s parameter count, computational load, and size, different pruning strategies were applied to the improved network. The global pruning method Lamp (Leveraging Adaptable Model Pruning) [27] is similar to magnitude-based layer pruning but features automatically selected sparsity levels. Pruning with Lamp retains the benefits of Magnitude Pruning (MP) while providing more flexibility and efficiency. Additionally, the computation of the Lamp score is efficient, does not require hyperparameters, and is independent of any model-specific knowledge. Ultimately, a pruning method based on Lamp scores was selected. The LAMP score is used for channel pruning in the improved network model, and this method does not require complex operations. It only requires adjusting the acceleration ratio. The specific formula is as follows:

(7) $$S\left(u;W_{t}\right)=\frac{(W_{t}[u]{)}^{2}}{\sum_{\nu ⩾u}\;(W_{t}[\nu ]{)}^{2}}$$

(8) $$\left(W\;[u]{)}^{2}>(W\;[v]{)}^{2}\Rightarrow score(u;W)>score(v,W\right)$$

where W denotes the weight, the numerator part $(W\;[v]{)}^{2}$ denotes the square of the weight magnitude of the target connection, and the denominator part $\sum_{\nu ⩾u}\;(W_{t}[\nu ]{)}^{2}$ denotes the sum of squares of the weight magnitudes of all the remaining connections in the same 1-layer. The weights are sorted in ascending order according to the given index mapping. According to Equation (7), it can be seen that the larger the weight term, the larger the corresponding LAMP score.

**Figure 6:**
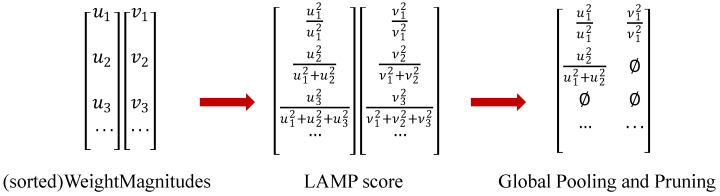
Lamp pruning flow chart.

The main process of LAMP score pruning is as follows: Initialize the weights from the base model after training. Calculate the squared magnitude of the connected weights and normalize it to obtain the sum of squared magnitudes of all weights in the same layer, which is the LAMP score. Based on the LAMP score, prune the connections by selecting the appropriate number according to the pre-set acceleration ratio. To avoid model collapse and detection failure, retain at least one channel in each layer. Fine-tune the pruned model to recover any performance loss during the pruning process. The pruning method using LAMP scores is illustrated in Figure 6.

## 3. Results

### 3.1. Training Results and Analysis of YOLOv8-P2 Model

After 300 training epochs, the YOLO v8-P2 model achieved the following results on the test set: precision (P) of 89.1%, recall (R) of 87.8%, mean average precision (mAP) of 91.2%, Params of 2.92 M, FLOPs of 12.2 G, and model size (MS) of 6.2 MB. The detailed detection results for each part are shown in Table 4.

In order to verify the validity of the YOLO v8-P2 model, the mean precision rate and mean recall rate were analyzed for different categories of calf behaviors. The results showed that the best performance of the different parts according to the precision rate was 96.1% for drinking precision. In comparison, standing precision was 74%, performing poorly. The best performance by recall was achieved for lying down with 99.2%, while walking achieved 56%, performing poorly. The best performance by mAP was achieved for drinking and lying down with 98.1%; in comparison, walking achieved 67.9%, performing poorly. The reason for these analysis results is that the lying behavior of cattle, with all limbs and the body lying on the ground, has obvious features and is easy to identify; furthermore, the drinking and eating behaviors are located in specific areas, with obvious features, and are easy to identify. Meanwhile, the difference between the standing behavior and the walking behavior lies in the cow’s head-twisting posture and leg-twisting posture, which are not easy to differentiate and easy to confuse, resulting in a lower recognition precision.

### 3.2. Pruning Strategy Comparison Experiment and Analysis

Deep neural networks are currently showing superior performance in various fields. However, their consumption of computational power and memory is also prohibitive. In order to utilize the advantages of large models in limited hardware resources, the application of pruning algorithms has received more and more attention from the public. The main influence on the results of channel pruning is related to the acceleration ratio (speed-up, sp) and sparsity rate (sparsity rate, sr). To ensure the reasonableness of the experiment, the variables in this experiment were the acceleration ratio and sparsity rate, and the rest of the variables were default values. Since the Silm strategy involves sparse training, 500 rounds of sparse training are required, followed by 250 rounds of channel pruning training. To find the optimal model accuracy for different speed-up ratios (sp) and sparsity rates (sr), this study referred to the parameter configuration from the literature and adjusted the value of sr based on preliminary experimental data. The values for sr were set to 0.0005, 0.0001, 0.005, 0.001, 0.05, and 0.01, and sparse training was performed on the improved model.

**Figure 7:**
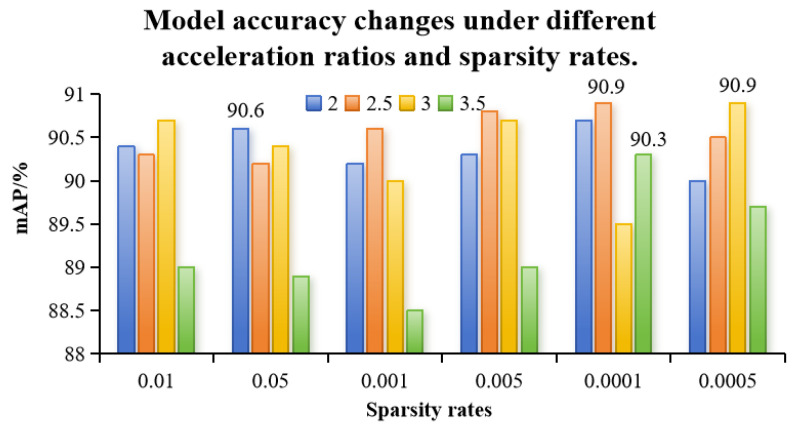
Changes in model accuracy for different acceleration ratios and sparsity rates. 90.6 is the highest mAP at 2, 90.9 is the highest at 2.5, 90.3 is the highest at 3, and 90.9 is the highest at 3.5.

In order to find the optimal model accuracy for different acceleration ratios sp and sparsity rates sr, this study referred to the parameter configurations in the literature [28], and based on the data from the pre-experimentation, the values for sr were set to 0.0005, 0.0001, 0.005, 0.001, 0.05, and 0.01 after sparsification training for the improved model. The model accuracy was the highest (90.9%) when the acceleration ratio sp was 2.5 and the sparsity rate sr was 0.05 and when the acceleration ratio sp was 3.0 and the sparsity rate sr was 0.01, as shown in Figure 7.

To validate the lightweight effect of the improved model, a comparative study was conducted using the C2f-Faster module fused with the EMA attention mechanism, Lamp, Slim (Network Slimming) [29], Random [30], and DepGraph [31] pruning techniques. The pruning ratios of the weight parameters before and after pruning were set to 2.5 and 3.0, respectively. The C2f-faster module replaces the Bottleneck module in C2f with the FasterBlock module from FasterNet and incorporates the EMA attention mechanism. This mechanism reduces the model’s computational load and parameter count by learning across spatial dimensions to fuse the output features of two parallel sub-networks. Lamp pruning involves trimming connections with smaller Lamp scores. Slim pruning determines the channel importance based on the scaling factor γ in the BN layer, retaining channels with larger values. Random pruning involves randomly trimming parameters. DepGraph is a non-depth graph algorithm based on a Dependency Graph (DepGraph), which implements a structurally universal pruning scheme. It allows for automatic parameter grouping and effectively enhances the generalization of structured pruning across various network architectures. Table 5 presents the experimental results of the models.

In Table 5, the improved algorithm incorporating the P2 module is abbreviated as V8-P2. As shown in Table 4, the V8-P2-Slim model suffers a relatively large loss in accuracy compared to the other models. This might be due to the pruning of redundant channels during the sparse training process, which leads to the model losing too much important information. The C2f-faster-EMA model has an mAP value similar to that of the other models, but its model parameters, floating point operations, and model size are relatively larger, making its lightweight effect less ideal. The Lamp, Random, and DepGraph pruning strategies perform best in terms of lightweighting when the speed-up ratio is 3.0 and the sparsity rate is 0.01. Among them, the Lamp strategy achieves the highest mAP value. The experiment shows that pruning connections with smaller Lamp scores can reduce computational costs without causing sparse connection issues.

The Lamp pruning strategy reduces the number of parameters by 68.4% through global sparsification, but the mAP only decreases by 0.3. This suggests that the removal of redundant channels has less impact on model performance and the computational efficiency improvement is more applicable to real-time deployment scenarios.

### 3.3. Comparison of Performance of Different Network Models

To evaluate the model’s detection performance for calf daily behaviors, this study conducted a performance assessment on eight models: SSD [32], YOLO v5n, YOLO v8n, YOLO v8-C2f-faster-EMA, YOLO v11n, YOLO v12n, YOLO v8-P2, and YOLO v8-P2-Lamp. All models were trained for 300 epochs on the same training and test datasets. The detailed results are presented in Table 6.

As shown in Table 6, the SSD and YOLO v5n models demonstrated inferior performance in calf behavior recognition and exhibited relatively large model sizes. Among the remaining six models, the differences in accuracy were minimal, but significant variations were observed in terms of the number of parameters, floating point operations, and model sizes. The YOLO v8-C2f-faster-EMA, YOLOv11, and YOLOv12 models showed comparable parameter counts, achieving an approximately 20% lightweight performance improvement at the cost of a 1% reduction in the mAP compared to the baseline YOLO v8 model. The YOLO v8-P2-Lamp model attained an mAP of 90.9% with extremely low computational costs. The YOLO v8-P2 model achieved the highest mAP of 91.2% but required 12.2 GFLOPs.

Based on this comprehensive comparison of the various models, the model developed in this study demonstrates significant practicality. It is lighter in terms of hardware and computational resource usage, providing strong technical support for mobile terminal applications.

**Figure 8:**
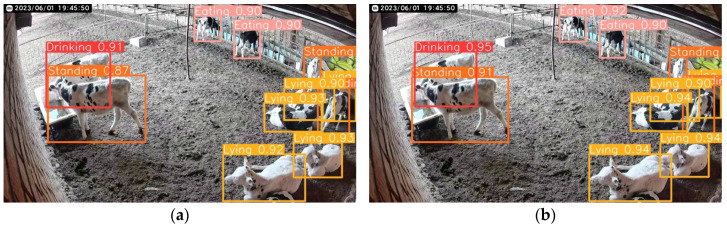
Behavioral recognition effects of different models of cows in the same scene. (**a**) YOLO v8n detection results; (**b**) YOLO v8-P2-Lamp test results.

To validate the real-world detection performance, a comparison between YOLO v8n and YOLO v8-P2-Lamp was conducted. Figure 8 presents the detection results for calf daily behaviors.

## 4. Discussion

In this study, a P2 small-target detection layer is introduced into the YOLOv8n model to enhance the high-resolution input scene and significantly improve the model recognition accuracy. Parameters or channels that have less impact on the output are selectively removed by LAMP pruning, thus reducing the computational complexity and storage requirements of the model, and improving the detection efficiency while maintaining the performance. However, due to the complexity of the farm environment, calf behavior data often have different light intensities and shading levels, as well as other interference factors, and these problems may lead to a reduction in calf behavior detection accuracy.

**Figure 9:**
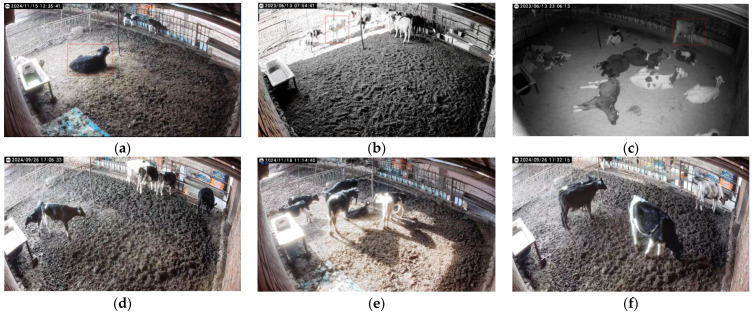
Schematic diagrams of different light and shading levels. (**a**) Calf in a daytime scene; (**b**) calf in a daytime (exposure) scene; (**c**) calf in a nighttime scene; (**d**) calf in a light-occlusion scene; (**e**) calf in a moderate-occlusion scene; (**f**) calf in a heavy-occlusion scene.

Figure 9a–c show some examples of calf behaviors under different light intensities, where the calf image is clearly visible during the day, while the target calf area is too bright or too dark in the exposure and nighttime environments, which results in an unclear image of the target calf and interferes with the model judgment. Due to the calf’s living enclosure being smaller, there is severe shading of the calf. As shown in Figure 9d–f, due to the smaller feeding trough, the calves are more crowded when feeding, and there is a heavy degree of occlusion, making it is difficult for the model to distinguish individual calves when the cows are dense, which leads to a reduction in the recognition accuracy.

To analyze the impact of the lighting intensity and occlusion on the model’s recognition performance, this study selected a subset of images categorized by lighting and occlusion conditions. The F1 score and mAP were used as evaluation metrics to analyze six calf behavior detection models.

### 4.1. Impact of Lighting Intensity on Model Performance

Calf behavior detection relies on the acquisition of data from cameras installed at fixed locations; however, changes in lighting conditions (e.g., overexposure or underexposure) can significantly affect the clarity of calf behavior, which in turn affects the detection performance of the model. To further analyze the performance of different models under different lighting conditions, this study conducted comparative experiments on six algorithms, and the results are shown in Table 7.

Lighting conditions had a significant effect on the performance of the calf behavior detection models. The models all inevitably showed accuracy degradation, and the average F1 values and mAPs of all the models under the three lighting conditions of daytime, daytime (exposure), and nighttime were 86.3, 84.5, and 82.5 and 89.3%, 86.1%, and 83.9%, respectively; the three models with the best performances were YOLOv8n, YOLOv8-P2, and YOLOv8n. This suggests that YOLOv8n is able to better capture the behavioral characteristics of calves under daytime conditions with higher detection accuracy. Overexposure leads to the loss of image details, which in turn affects the detection accuracy of the model, while YOLOv8-P2 is the model with the lowest loss of accuracy under the exposure environment. Insufficient light at nighttime leads to increased image noise and blurring of the target area, which in turn affects the detection accuracy of the model. YOLOv8n is the model with the best anti-interference ability at night. YOLO v8-P2-Lamp shows a higher effect than the overall average mAP during daytime and nighttime, and the anti-interference ability is weaker under exposure conditions. The stability of the model should be improved subsequently for overexposure, and the overall stability is weaker than that of YOLOv8n and YOLOv8-P2 for the lightweight model.

### 4.2. Impact of Occlusion Degree on Model Performance

In addition to lighting conditions, calf behavior detection is challenged by the problem of shading. In the farm environment, body contact between calves, cross-obscuration, and occlusion from facilities such as feed troughs can affect the detection accuracy of the model. To further analyze the performance of different models under different levels of occlusion, this study conducted comparative experiments on six algorithms, and the results are shown in Table 8.

The degree of masking had a significant effect on the performance of the calf behavior detection models. The average F1 values and mAPs of the models at different occlusion levels were 84.9, 88.5 and 89.3%, 86.1%, and 78.5%, 82.1%, respectively, and the three models with the best performances were YOLOv8n, YOLO v8-C2f-faster-EMA, and YOLO v8-P2-Lamp. This suggests that the YOLOv8n model is able to better deal with light degrees of occlusion and maintains high detection accuracy. YOLO v8-C2f-faster-EMA can effectively enhance the feature extraction ability for occluded targets by introducing the C2f-faster module and EMA attention mechanism, thus maintaining high detection accuracy under moderate-occlusion conditions; heavy occlusion leads to the loss of target features, which in turn affects the detection accuracy of the model. In this case, the accuracy of all models is severely degraded. YOLO v8-P2-Lamp can recognize subtle features by introducing a small-target detection layer and remove redundant channels through the Lamp strategy, thus maintaining high detection accuracy under heavy-occlusion conditions. Therefore, in practical applications, different models can be selected for monitoring by the size of cattle farms.

### 4.3. Visualization Methods

Grad-CAM is a gradient-based localization method for visualizing deep neural networks and demonstrating features obtained through convolutional networks. The method consists of computing the weights of each feature map, deriving the global average gradient, and performing backpropagation to obtain the gradient values. This method helps to analyze the focus area of the network about a particular class, which is used to assess whether a particular feature or piece of information has been accurately obtained. In the heat map, darker shades of red indicate that the region contributes more to the final prediction, indicating that the network has increased its focus on this part of the image. Conversely, a darker blue color indicates a reduced contribution to the final prediction, meaning that the network considers the information to be redundant.

**Figure 10:**
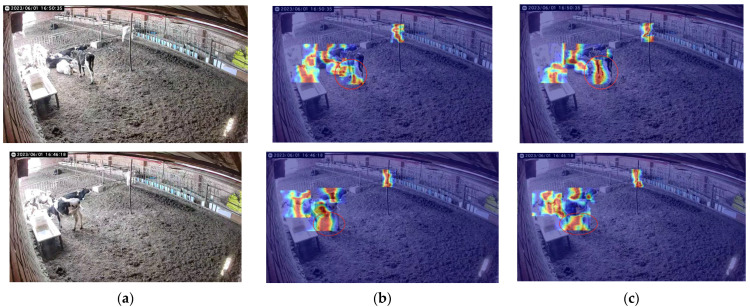
Heat map visualization comparison. (**a**) Original figure; (**b**) heat map of YOLOv8n; (**c**) heat map of YOLOv8n-P2. The red circle highlights the area for visual detail comparison.

In order to further reflect the superior performance of the models in this chapter, this section visualizes the heat maps of the feature maps of the prediction results of YOLOv8n and the algorithms in this chapter, as shown in Figure 10, and it is not difficult to see that Figure 10c is more accurate concerning the details of the calf’s legs in the input image and contributes more to the detection decision compared to Figure 10b.

### 4.4. Limitations and Future Directions

The lightweight model introduced in this paper still has some limitations in calf behavior recognition. First, the dataset used for validation is relatively small, and the behavior categories are not diverse enough, lacking broader data support. Therefore, future experiments need to be conducted on larger datasets to more comprehensively assess the performance of the lightweight model. Second, the design approach of the lightweight model in this study is not comprehensive, as it does not incorporate some emerging technologies (such as adaptive computing). This will be an important direction for future improvements. Finally, the training and deployment processes of the lightweight model remain somewhat complex, and more intelligent and automated tools are needed to simplify operations.

In this study, the model’s accuracy in calf behavior detection was reduced when interference factors such as different lighting conditions and varying degrees of occlusion were present. Future work could consider using image enhancement techniques or infrared cameras for preprocessing, integrating multi-angle cameras, or incorporating contextual information to improve the model’s performance in detecting calf behavior in complex environments.

The current model has limited ability to recognize low-frequency behaviors such as early signs of disease. In the future, it can be combined with fine-grained classification networks such as ResNeSt or temporal anomaly detection algorithms such as LSTM-Attention to improve the sensitivity to subtle behavioral changes.

This study validated the model’s performance in a small- to medium-sized farm environment, which needs to be scaled up to large-scale farms in the future, along with optimization of the camera deployment scheme for high-density farming scenarios.

Future plans are to introduce additional behavioral categories and calf types, and to expand extreme weather scenarios through data augmentation to improve the model’s generalizability.

In the future, the performance of the model will be tested for deployment in edge devices, and quantization or TensorRT acceleration techniques will be explored.

## 5. Conclusions

(1) This study introduces a P2 small-object detection layer into the YOLO v8n network, proposing an improved YOLOv8-P2 network model. After the improvement, the network model achieved a precision of 89.1%, recall of 87.8%, and mean average precision (mAP) of 91.2%, with 2.92 M parameters, 12.2 G FLOPs, and a model size of 6.2 MB. The precision, recall, and mAP were all improved, and the parameter count was significantly decreased. This demonstrates that the introduced modifications effectively enhanced the model’s performance.
(2) In this study exploring how model performance is affected by different sparsity rates and speed-up ratios, the experimental results show that the pruned model performed optimally when the sparsity rate was set to 0.0005 and the speed-up ratio was set to 3.0. Under these conditions, the model’s mean average precision (mAP) reached 90.9%, the parameter count was reduced to 0.949 M, the computational load decreased to 4.0 GFLOPs, and the model size was compressed to 2.3 MB. Compared to the original YOLO v8n network, the parameter count was reduced by 68.4%, the computational load decreased by 35.5%, and the model size was reduced by 62.9%, while the network’s mAP increased by 0.7%. This effectively reduced the model size and improved the network’s accuracy.
(3) Compared with the SSD, YOLO v5n, YOLO v8n, YOLO v8-C2f-faster-EMA, and YOLO v8-P2 models, the model proposed in this study increased the average precision (mAP) by 0.7% while significantly reducing the number of parameters, the amount of computation, and the model size. This indicates that the pruning technology used can effectively enhance the performance of the model and provide solid technical support for real-time and accurate monitoring of the daily behavior of calves in breeding farms and deployment in mobile devices with less hardware and computational resources.

## Figures and Tables

**Table 1:** Main contributions of this paper.

Sequences	Main Contributions
1.	An improved model of YOLOv8 incorporating the P2 small-target detection layer is proposed, which significantly improves the recognition accuracy of small targets (e.g., calf leg bending).
2.	The Lamp pruning strategy is introduced to reduce the number of model parameters and computational cost while maintaining a high mAP.
3.	A calf behavior dataset containing complex lighting and occlusion scenarios is constructed, and the robustness of the model is verified.

**Table 2:** Criteria for determining cow behaviors.

Type	Behavioral Description	Labels
Walking	Alternated bending of limbs, trunk horizontal, head raised	Walking
Standing	Leg upright to support body	Standing
Lying Down	Abdominal contact with ground	Lying
Feeding	Feeding with head through railing	Eating
Drinking Water	Drinking with head over sink	Drink

**Table 3:** Training parameter setting.

Parameter	Values
Training batch size	16
Epochs	250
Image size	640 × 640
Batch	128
Initial learning rate	0.01
Momentum	0.937
Sparsity training epochs	500
Parameter	Values

**Table 4:** Comparison of test accuracy for different behaviors.

Behavior	Precision (%)	Recall (%)	mAP_50_ (%)
Drinking	79.2	78.3	78.7
Lying	76.0	77.3	79.0
Eating	89.3	89.3	91.5
Standing	93.1	90.0	94.0
Walking	93.8	96.7	97.7

**Table 5:** Model lightweight ablation experiments.

Index Algorithms	Params (M)	FLOPS (G)	Model Size (Mb)	Precision (%)	Recall (%)	mAP50 (%)
YOLO v8-C2f-faster-EMA	2.310	6.5	4.9	88.5	85.6	89.5
V8-P2-Lamp/2.5/0.0001	1.103	4.5	2.6	89.0	85.6	90.9
V8-P2-Lamp/3.0/0.0005	0.949	4.0	2.3	88.1	87.6	90.9
V8-P2-Slim/2.5/0.0001	2.431	4.9	5.2	85.4	84.9	87.5
V8-P2-Slim/3.0/0.0005	2.320	4.0	5.0	85.7	85.9	87.9
V8-P2-Random/2.5/0.0001	1.103	4.5	2.6	90.0	85.0	90.0
V8-P2-Random/3.0/0.0005	0.949	4.0	2.3	86.1	88.6	90.4
V8-P2-DepGraph/2.5/0.0001	1.103	4.5	2.6	89.1	86.5	89.8
V8-P2-DepGraph/3.0/0.0005	0.949	4.0	2.3	89.9	85.8	90.3

**Table 6:** Multi-model performance comparison.

Index Algorithms	Params (M)	FLOPS (G)	Model Size (Mb)	Precision (%)	Recall (%)	mAP50 (%)
SSD	52	26.2	92.6	87.1	69.2	84.5
YOLO v5n	3.2	4.6	27	86.8	68.7	80.9
YOLO v8n	3.007	8.1	6.2	88.7	87.6	90.2
YOLO v8-C2f-faster-EMA	2.310	6.5	4.9	88.5	85.6	89.5
YOLO v11n	2.583	6.3	5.5	87.4	86.0	89.2
YOLO v12n	2.569	6.5	5.3	87.9	84.8	89.0
YOLO v8-P2	2.922	12.2	6.2	89.1	87.8	91.2
YOLO v8-P2-Lamp	0.949	4.0	2.3	88.1	87.6	90.9

**Table 7:** Experimental results of six models under different lighting conditions.

Category	Number	Index Algorithms	F1 Score (%)	mAP50 (%)
Daytime	2525	SSD	83	85.6
		YOLO v5n	85	87.0
		YOLO v8n	88	91.4
		YOLO v8-C2f-faster-EMA	86	89.8
		YOLO v8-P2	89	91.1
		YOLO v8-P2-Lamp	87	90.8
Daytime (exposure)	2526	SSD	83	82.1
		YOLO v5n	85	85.4
		YOLO v8n	87	88.0
		YOLO v8-C2f-faster-EMA	84	87.2
		YOLO v8-P2	87	88.7
		YOLO v8-P2-Lamp	81	85.1
Nighttime	3007	SSD	79	82.4
		YOLO v5n	81	83.0
		YOLO v8n	86	85.4
		YOLO v8-C2f-faster-EMA	85	83.1
		YOLO v8-P2	84	84.5
		YOLO v8-P2-Lamp	80	84.8

**Table 8:** Experimental results of the five models under different shading levels.

Category	Number	Index Algorithms	F1 Score (%)	mAP50 (%)
Light masking	2589	SSD	83	81.3
		YOLO v5n	85	83.5
		YOLO v8n	87	88.5
		YOLO v8-C2f-faster-EMA	84	87.7
		YOLO v8-P2	87	88.6
		YOLO v8-P2-Lamp	83	86.0
Medium masking	2413	SSD	82	80.3
		YOLO v5n	86	82.3
		YOLO v8n	91	92.3
		YOLO v8-C2f-faster-EMA	92	92.9
		YOLO v8-P2	91	92.7
		YOLO v8-P2-Lamp	89	92.3
Heavy masking	2379	SSD	73	82.1
		YOLO v5n	79	83.6
		YOLO v8n	81	82.9
		YOLO v8-C2f-faster-EMA	75	77.7
		YOLO v8-P2	83	82.9
		YOLO v8-P2-Lamp	80	83.5

## Data Availability

The data are not publicly available due to being part of an ongoing study.
